# Positive affect, childhood adversity, and psychopathology in psychiatric inpatients

**DOI:** 10.3402/ejpt.v4i0.20771

**Published:** 2013-08-12

**Authors:** Darryl W. Etter, Justin R. Gauthier, Elizabeth McDade-Montez, Marylene Cloitre, Eve B. Carlson

**Affiliations:** 1 PGSP-Stanford Psy.D. Consortium, Palo Alto University, Palo Alto, CA, USA; 2 Department of Counseling, Clinical, and School Psychology, University of California, Santa Barbara, CA, USA; 3 Department of Psychology, University of California, Merced, CA, USA; 4 National Center for PTSD, VA Palo Alto Health Care System, Palo Alto, CA, USA

**Keywords:** childhood adversity, positive affect, sexual abuse, social support, trauma

## Abstract

**Background:**

Low positive affect is closely related to common pathological responses to childhood adversity, including posttraumatic stress disorder (PTSD) and depression, but little is known about how the characteristics of early adversity experiences might be related to positive affect in adulthood.

**Objective:**

This study aimed to explore whether low positive affect is related to specific childhood adversities, including abuse, neglect, caretaker dysfunction, and low childhood social support.

**Method:**

Using structured interviews and self-report measure data collected from 173 adult psychiatric inpatients, this study examined the relationship between positive affect and symptoms of psychopathology, as well as how the number of types of abuse experienced, severity of adversity types (physical abuse and sexual abuse), childhood environment (childhood social support, neglect, and caretaker dysfunction), and number of non-abuse traumas related to positive affect.

**Results:**

Positive affect was significantly negatively related to several symptoms of psychopathology, including depression, dissociation, self-destructive behavior, PTSD, and global psychopathology. Individuals who experienced both physical and sexual abuse reported significantly less positive affect than those with only physical or no abuse experiences. Lower positive affect was predicted by lower childhood social support and greater severity of sexual abuse, with both factors accounting for unique variance in positive affect.

**Conclusion:**

These results suggest that individuals who experience multiple types of early adversity, more severe sexual abuse experiences, and less social support are at risk of psychological difficulties. Given the relatively strong association between positive affect and childhood social support, interventions to foster social support may be a means of increasing positive affect among individuals exposed to childhood adversity.

Low positive affect is an important component of psychopathology that is associated with poorer functioning and resilience to daily and extreme stressors, yet little research has investigated its relationship with traumatic stressors, including childhood adversity. Better understanding the affective experiences associated with childhood adversity could ultimately allow for the development of more effective interventions for individuals who experienced early adversities, including fostering positive affect to promote resilience to and recovery from these stressors. This study aimed to explore whether low positive affect is related to specific childhood adversities, including abuse, neglect, caretaker dysfunction, and low childhood social support.

## Low positive affect and psychopathology

Low positive affect is an important component of various forms of psychopathology, including social anxiety and depression (Naragon-Gainey, Watson, & Markon, 2009; Watson, Gamez, & Simms, 2005), and has been associated with posttraumatic stress disorder (PTSD) and self-harm (Frewen et al., 2010; Gratz, 2006). Low positive affect is conceptually (Watson et al., 1995) and neurologically (see Davidson, 2000) distinct from negative affect. Therefore, low positive affect presents an additional challenge to individuals’ functionality with distinctive psychological, cognitive, and neurological correlates. Lower positive affect is associated with greater PTSD symptom severity (Feeny, Zoellner, Fitzgibbons, & Foa, 2000) and poor recovery from depressive episodes (Rottenberg, Kasch, Gross, & Gotlib, 2002). Individuals with low positive affect also have worse memory for positive events, expect fewer future positive events, and may have self-regulation difficulties that limit their ability to achieve goals (Andersen & Limpert, 2001; Aspinwall, 1998). Neurologically, low positive affect has been associated with decreased activation in brain areas involved in reward and higher-order cognition (Forbes & Dahl, 2005; Frewen et al., 2010).

In contrast, greater positive affect seems to be an important factor in resilience to and recovery from psychological stressors, including depressive and PTSD symptoms (Ong, Bergeman, Bisconti, & Wallace, 2006; Yamasaki, Uchida, & Katsuma, 2010). The NIMH Research Domain Criteria (RDoC) project has identified positive affect as an important area of functioning, and dysfunction of this system is considered to cut across diagnoses (Sanislow et al., 2010). Better characterizing the association between positive affect and traumatic stressors, including childhood adversities, could inform our understanding of the factors involved in the development and maintenance of posttraumatic psychopathology, as well as prevention of and recovery from these disorders.

## Childhood adversities and posttraumatic outcomes

Rather than being considered as a specific outcome, low positive affect in adulthood has been primarily tied to childhood adversity through its associations with depression and PTSD (Green et al., 2010; Hillberg, Hamilton-Giachritsis, & Dixon, 2011). However, low or inappropriate positive affect has also been directly associated with poorer functioning in childhood sexual abuse survivors (Bonanno et al., 2007), and greater severity of childhood abuse experiences has been associated with lower positive affect among women with PTSD (Frewen, Dozois, & Lanius, 2012). The nature of the relationship between positive affect and specific adversities relative to other life experiences remains unclear, though. Based on studies of the association between childhood adversity and other negative psychological outcomes, low positive affect in adulthood may be associated with the severity and type of adversity experienced.

The severity of childhood adversity is often measured by the number of discrete maltreatment experiences or by the number of types of adversity experienced. Experiencing more incidents of maltreatment has been associated with greater levels of PTSD and depression symptoms (Vranceanu, Hobfoll, & Johnson, 2007). Similarly, experiencing multiple types of maltreatment has consistently been found to be related to poorer psychological functioning in adulthood than experiencing only one type of maltreatment (Cloitre et al., 2009; Edwards, Holden, Felitti, & Anda, 2003; Green et al., 2010). Low positive affect will, therefore, likely be associated with greater severity of childhood adversity.

In contrast to the consistent association of severity of childhood adversity with poorer psychological outcomes, there is no clear consensus as to which types of adversity are most associated with psychopathology (Arata, Langhinrichsen-Rohling, Bowers, & O'Farrill-Swails, 2005; Paolucci, Genuis, & Violato, 2001). Recent findings of associations between multiple types of abuse and positive affect (Frewen et al., 2012) did not test for differences between types of abuse, but early case studies demonstrating differential effects of physical and emotional abuse on infants’ affect (Gaensbauer & Hiatt, 1984) suggest that examining the relationship between various types of childhood adversity and low positive affect may inform our understanding of how such experiences impair functioning.

Physical abuse, sexual abuse, and family environment (e.g., childhood social support, neglect) have all been associated with adulthood psychopathology (e.g., Edwards et al., 2003), and the strength of those associations can inform expectations about the relationships between adversities and low positive affect. Relative to some other early adversities, sexual abuse has been found to be more predictive of poor psychological adjustment in adulthood, PTSD symptoms, and negative affect (Higgins & McCabe, 2000; Sullivan, Fehon, Andres-Hyman, Lipschitz, & Grilo, 2006). Lower levels of childhood social support, especially emotional and psychological support, have been associated with PTSD, emotional distress, depression, negative affect, externalizing symptoms, and lower levels of psychological wellbeing (Lauterbach, Koch, & Porter, 2007; Sullivan et al., 2006). The role of physical neglect is less clear because it so often co-occurs with other types of early adversity. Some evidence suggests that it may be less predictive of PTSD, depressive, and dissociative symptoms than physical or sexual abuse (Wechsler-Zimring & Kearney, 2011). Positive affect is an important aspect of psychological functioning and wellbeing that is potentially affected by these same early adversities.

## The current study

Low positive affect is closely related to common pathological responses to childhood adversity, including PTSD and depression, but little is known about how the characteristics of early adversity experiences might be related to positive affect in adulthood. Therefore, this study examined how the number of types of abuse experienced, severity of adversity types (physical abuse and sexual abuse), childhood environment (childhood social support, neglect, and caretaker dysfunction), and number of non-abuse traumas are related to positive affect. First, we hypothesized that lower positive affect would be associated with multiple types of abuse (i.e., both physical and sexual abuse). Second, we hypothesized that sexual abuse would be more strongly related to low positive affect than physical abuse. Third, we expected that lower positive affect would be related to lower levels of childhood social support, higher levels of neglect, higher levels of caretaker dysfunction, greater severity of abuse, and more non-abuse traumas. Finally, we sought to replicate earlier findings of associations between low positive affect and other psychiatric symptoms.

## Method

### Participants and procedure

We conducted a secondary analysis of data collected from psychiatric inpatients participating in a study examining the relationship of childhood family environment, childhood abuse experiences, and adult traumatic stress with current posttraumatic symptoms (Carlson et al., 2001). A detailed description of the recruitment methods is described elsewhere (Carlson et al., 2001). All patients who were admitted to a large, nonprofit psychiatric hospital over a period of 3.5 years were eligible to participate in the study and patients were targeted for recruitment as soon as approval from the admitting psychiatrist or psychologist was obtained. Two hundred seventeen adults participated in a structured clinical interview, and data for all predictors were available for 173 participants. The majority of participants were female (52%), and most participants reported their ethnicity as Caucasian (78.6%) or African-American (18.7%). Mean age was 37.7 years (*SD*=5.0). Adult socio-economic status (SES) was determined using Hollingshead's Two-Factor Index of Social Position (Hollingshead & Redlich, 1958), which is based on education level and occupation of the participant or the head of household (whichever results in higher SES). In this sample, 8% of participants were categorized as lower class, 29% as lower-middle class, 26% as middle class, 27% as upper-middle class, and 10% as upper class.

Psychiatric diagnoses were obtained from patient charts. The most common diagnoses were mood disorders, followed by substance use disorders (see Table 1). Other data were collected through self-report measures and private interviews by trained and supervised clinical psychology graduate students who were not involved in the participant's treatment.

**Table 1:** Prevalence of psychiatric diagnoses

Disorder type	Percent diagnosed
Mood	58.4
Substance use	32.9
Dissociative	22.5
Anxiety	19.7
Others	15.0
Psychotic	6.4
Eating	4.0
Borderline	3.5

### Measures

#### Positive affect

Positive affect was assessed with two interviewer-administered questions. First, participants responded to an item describing their perceived capacity for positive affect (“In general, over your whole life, how easy or hard has it been for you to feel good or happy?”) on a 6-point Likert scale (0*=extremely hard*; 1=*very hard*; 2=*a little bit hard*; 3=*fairly easy*; 4=*very easy*; 5=*extremely easy*). Second, participants responded to an item describing their experience of positive affect (“In general, over your whole life, how often have you felt good or happy?”) on a 6-point Likert scale (0=*never*; 1=*almost never*; 2=*sometimes*; 3=*fairly often*; 4=*often*; 5=*very often*). Summing the two item scores created an index of positive affect capacity and frequency. Positive affect index scores could range from 0 to 10. In this sample, the two items were significantly correlated, *r=*0.59, *p*<0.001.

#### Global psychopathology symptoms

The Symptom Checklist-90-Revised (SCL-90-R; Derogatis, 1983) was used to measure global psychopathology symptoms. The SCL-90-R is a 90-item self-report questionnaire that measures nine psychiatric symptoms as well as the overall degree of distress related to a symptom with a 5-point scale (0=*not at all*; 1*=a little bit*; 2*=moderately*; 3*=quite a bit*; 4=*extremely*). Subscale scores are averages of subscale items, and the Global Severity Index represents global psychopathology symptoms by averaging subscale scores. The SCL-90-R has been well validated for use in psychiatric samples (Derogatis, 1983).

#### Depression symptoms

The Depression subscale of the SCL-90-R was used to assess depression symptoms. Though this scale is included in the SCL-90-R total score, it was included in bivariate correlation analyses due to the specific association between positive affect and depression in prior research (Watson et al., 2005).

#### Dissociative symptoms

Dissociative symptoms were measured with the Dissociative Experiences Scale (DES; Bernstein & Putnam, 1986). It is a 28-item self-report measure assessing the percentage of time individuals experience amnesia, depersonalization, derealization, absorption, and imaginative involvement. The items are averaged for a total score, with higher scores indicating more dissociative symptoms. The DES is widely used and has strong psychometric properties (Carlson & Putnam, 1993; Waller, 1995).

#### Self-destructive behaviors

The Structured Interview of Self-Destructive Behaviors (SI-SDB; Carlson, McDade-Montez, Dalenberg, Armstrong, & Loewenstein, 2013) assesses the lifetime level of self-destructive behaviors of suicidality, self-injury, risky sexual behaviors, disordered eating, and substance abuse. Analyses of internal and external validity are supportive of the validity of the measure (Carlson et al., 2013). The interviewer rates the severity of impairment in each domain, where 0=*none*, 1=*mild*, 2=*moderate* and 3=*severe*. Scores for each domain are summed for a total score ranging from 0 to 15.

#### PTSD symptoms

The Structured Interview for PTSD (SI-PTSD; Davidson, Smith, & Kudler, 1989) rates the severity of the 17 symptoms of Criteria B, C, and D for PTSD in the DSM-IV-TR. The SI-PTSD uses a screening item to identify a traumatic event, and the interview is only completed if a traumatic event is reported. For this reason, SI-PTSD scores were available for 133 out of 173 participants. The SI-PTSD has demonstrated good interrater and test–retest reliability, as well as concurrent validity (Davidson et al., 1989).

#### Childhood social support

Perceived childhood social support was assessed with a measure created for the study, the Structured Interview for Social Support as a Child (SI-SSC). The SI-SSC assesses five types of support: self-esteem support, informational support, instrumental support, motivational support, and listening support. Participants indicated when and who provided the support, and the extent they found this person helpful was rated from 0 to 3, where 0=*not at all*, 1=*a little*, 2=*somewhat*, and 3=*very much*. Total scores were then calculated by averaging across all five items, for a possible range of 0–3. The SI-SSC has been shown to be reliable and valid (Carlson et al., 2001).

#### Neglect

Neglect was assessed with five questions as part of a structured interview, participants were asked whether they experienced being sick with no one to care for them, getting sick because of neglect, going without food or water for a day or more, living away from home for 3 or more months, or living in foster care. Scores represent how many of these types of experiences an individual reported and could range from 0 to 5.

#### Caretaker dysfunction

As part of a structured interview about childhood experiences, participants were asked with whom they lived as children and who was responsible for their care, up to five individuals. For each of these caretakers, participants indicated whether that person had a mental illness, had problems with drinking or alcohol abuse, had ever attempted suicide, or had been violent with others in the home. Scores represent how many of these issues were endorsed and could range from 0 to 20.

#### Childhood sexual and physical abuse

Childhood sexual and physical abuse were assessed with slightly modified versions of Jacobson's (1989); Jacobson & Richardson, 1987) structured interviews for interpersonal violence and nonconsensual sexual experiences which were based on the Conflict Tactics Scale (CTS; Straus, 1979). Participants were first asked “When you were a child, did anyone ever use physical force on you?” Participants could identify up to four assailants, and for each individual identified, interviewers asked structured questions about 11 types of physical violence (e.g., “Did this person ever throw something at you?”), as well as the frequency and duration of the incidents. The interview followed a similar structure in assessing sexual abuse. Participants endorsing the gate question (“When you were a child, did anyone ever touch you in a sexual way or do something sexual or weird that made you feel uncomfortable?”) were asked about the frequency and duration of 11 types of sexual experiences with up to four individuals. The interview was structured and worded to be specific and neutral to increase accuracy of recall. The interview procedure used for this study is described in greater detail elsewhere (Carlson et al., 2001).

Responses from these interviews were classified into physical abuse or sexual abuse, and individuals were categorized as having experienced No Abuse, Physical Abuse Only, Sexual Abuse Only, or Both Abuse Types. Physical abuse severity and sexual abuse severity scores were calculated by summing the number of abuse experiences within each category, with more severe incidents (e.g., being burned or threatened with a weapon, sexual penetration) counted as double. Scores on these variables represent the number of experiences of these types of abuse from up to four abusers, such that multiple types of abuse on one occasion results in a higher score for participants. The interview and scoring system used for this study have demonstrated strong reliability and validity through test–retest reliability and convergent validity analyses reported in the original publication from this project (see Carlson et al., 2001).

Abuse scores were transformed to reduce distortion by statistical outliers resulting from some participants endorsing very high levels of abuse. We transformed these scores using a 95^th^ percentile Winsorization, in which outliers beyond the 95^th^ percentile are replaced with the value at the 95^th^ percentile. Winsorization retains all data and their magnitudes and is more intuitively clear than other procedures, such as common log transformations (Sheskin, 2003).

#### Adulthood and childhood non-abuse trauma

The numbers of adulthood and childhood non-abuse traumatic events were collected as part of the SI-PTSD (Davidson et al., 1989). For this study, to avoid excluding those who had high levels of trauma, but were unable to provide exact numbers, the number of adulthood and childhood non-abuse traumas reported during the interview were coded into five categories where 0=0 traumas, 1=1 trauma, 2=2–3 traumas, 3=4–5 traumas, and 4=6 or more traumas.

## Results

### Descriptive and correlation analyses

Descriptive data for psychiatric symptoms and psychosocial factors are presented in Table 2. Positive affect was significantly negatively correlated with all other symptom measures (see Table 3). Table 4 shows correlations among positive affect, childhood environment (social support, caretaker dysfunction, and neglect), childhood physical abuse, childhood sexual abuse, childhood traumas, and adult traumas. Positive affect was significantly related to levels of childhood social support, caretaker dysfunction, physical abuse, and sexual abuse, but not to neglect, childhood non-abuse traumas, or adulthood non-abuse traumas.

**Table 2:** Mean levels of psychiatric symptoms and psychosocial stressors

			Range	
			
Variables	*M*	*SD*	Potential	Actual
Symptoms				
Positive affect	3.9	1.99	0–10	0–10
Global psychopathology	1.6	0.82	0–4	0.04–3.51
Depression	2.2	1.04	0–4	0–4
Dissociative symptoms	28.0	21.45	0–100	0–88.2
Self-destructive behavior	7.0	3.53	0–15	0–15
PTSD	27.2	16.46	0–68	0–60
Stressor			
Childhood social support	1.8	1.00	0–3	0–3
Neglect	1.1	1.38	0–5	0–5
Caretaker dysfunction	2.8	2.03	0–20	0–10
Physical abuse severity	8182.0	14318.26		0–57685.8
Sexual abuse severity	5126.7	12680.65		0–50746.9
Childhood non-abuse traumas	0.1	0.50	0–4	0–4
Adult non-abuse traumas	1.2	1.30	0–4	0–4

*Note*: *n*=173, except PTSD symptoms (*n*=133).

**Table 3:** Intercorrelations of positive affect and psychopathology symptoms

Symptoms	1	2	3	4	5	6
1. Positive affect		−0.42***	−0.37***	−0.35***	−0.42***	−0.43***
2. Global psychopathology			0.87***	0.57***	0.41***	0.59***
3. Depression				0.35***	0.36***	0.55***
4. Dissociative symptoms					0.35***	0.58***
5. Self-destructive behavior						0.40***
6. PTSD						

*Note*: *n*=173, except PTSD correlations (*n*=133).

*** *p*<0.001.

**Table 4:** Intercorrelations of positive affect and predictor variables

Predictors	1	2	3	4	5	6	7	8
1. Positive affect		0.43***	−0.12	−0.20**	−0.22**	−0.31***	−0.06	−0.08
2. Childhood social support			−0.07	−0.12	−0.26***	−0.24***	0.08	0.11
3. Neglect				0.36***	0.36***	0.32***	0.14	0.07
4. Caretaker dysfunction					0.36***	0.30***	0.09	0.03
5. Physical abuse severity						0.58***	0.04	0.04
6. Sexual abuse severity							0.17*	0.09
7. Childhood non-abuse traumas								−0.14
8. Adulthood non-abuse traumas								

* *p*<0.05

** *p*<0.01

*** *p*<0.001.

### Effect of number of types of abuse

To test our hypotheses regarding the association between type of abuse experienced and positive affect, an ANOVA was conducted on positive affect scores with abuse type (No Abuse, Physical Abuse Only, Sexual Abuse Only, Both Abuse Types) as the independent variable. The number of participants reporting each type of abuse and group means is presented in Table 5. This ANOVA found significant group differences, *F*(3,168)=8.1, *p*<0.001. Post-hoc comparisons with Bonferroni corrections showed that those in the Both Abuse Types group had significantly lower positive affect than those in the Physical Abuse Only and No Abuse groups. No other significant differences across groups were observed.

**Table 5:** Mean levels of positive affect by abuse type

Types of abuse	*n*	*M*	*SD*
No abuse	41	4.6^a^	1.9
Physical abuse only	61	4.5^b^	2.0
Sexual abuse only	12	3.5	1.4
Both abuse types	58	3.0^a,b^	1.7

*Note*: Groups with the same superscript differ at *p*<0.001.

### Effect of childhood adversity

To test the capacity of various types of childhood adversity to predict positive affect, a hierarchical regression analysis was conducted predicting positive affect with childhood environmental factors (social support, neglect, and caretaker dysfunction) entered in Step 1, severity of physical and sexual abuse entered in Step 2, and childhood and adulthood non-abuse traumas entered in Step 3. The results are shown in Table 6.

**Table 6:** Hierarchical multiple regression predicting positive affect

Predictors	Δ*R* ^2^	β
Step 1	**0.190** ***	
Childhood social support		0.41***
Neglect		−0.04
Caretaker dysfunction		−0.13
Step 2	**0.022** *	
Childhood social support		0.37***
Neglect		−0.01
Caretaker dysfunction		−0.10
Physical abuse severity		0.04
Sexual abuse severity		−0.21*
Step 3	**0.007**	
Childhood social support		0.40***
Neglect		0.01
Caretaker dysfunction		−0.10
Physical abuse severity		0.03
Sexual abuse severity		−0.18*
Childhood non-abuse traumas		−0.08
Adulthood non-abuse traumas		−0.12

*Note*: *R* ^2^ values are adjusted; β values are standardized.

* *p*<0.05

*** *p*<0.001.

## Discussion

This study demonstrated that positive affect is significantly negatively related to a number of psychiatric symptoms and that childhood support and abuse experiences are significant predictors of positive affect in adult psychiatric inpatients. Individuals who experienced both physical and sexual abuse reported significantly lower positive affect than those with only physical or no abuse experiences. Greater severity of sexual abuse and lower childhood social support predicted lower positive affect, above and beyond childhood neglect, caretaker dysfunction, physical abuse severity, and non-abuse trauma experiences.

We found positive affect to be negatively correlated with global psychopathology, depressive, dissociative, self-destructive behavior, and PTSD symptoms. These negative associations are consistent with the view that low positive affect is a transdiagnostic symptom. Our finding of associations with broad psychopathology may be a result of our assessment of positive affect broadly over the lifespan and measurement of a broad construct (i.e., happiness), whereas other more nosological studies measuring current affect have primarily associated low positive affect with depression (Watson et al., 2005). Positive affect is distinct from negative affect, such that one may experience high or low levels of either, both, or neither (Watson et al., 1995). Low positive affect, therefore, can cause distress and functional impairment independently or in conjunction with negative affect. Low positive affect may also increase vulnerability to psychological disorders by impairing cognitive processes involved in resilience to and recovery from negative psychological states (Andersen & Limpert, 2001; Aspinwall, 1998; Forbes & Dahl, 2005; Frewen et al., 2010).

The finding that individuals who experienced both types of abuse reported lower positive affect partially supported our hypothesis that multiple types of abuse would be associated with lower positive affect than one type or no abuse. This finding is also consistent with prior research on other outcomes that indicates that individuals who have experienced multi-type maltreatment may be at risk for compromised psychological functioning (e.g., Green et al., 2010). Interestingly, individuals reporting only one abuse type did not differ significantly from those reporting no abuse. However, the number of types of abuse experienced is a relatively imprecise index of severity of abuse, and the severity of the abuse in those with only one abuse type may have been lower than in those with more than one type.

The regression analyses sought to clarify the relationship between positive affect and the type and severity of early adversity. Severity of sexual abuse predicted significant unique variance in positive affect above and beyond childhood environment characteristics and non-abuse trauma experiences, while severity of physical abuse was not significantly predictive of positive affect. This provides support to our hypothesis that sexual abuse would be associated with lower positive affect and is consistent with prior research indicating that sexual abuse may be more pathogenic than physical abuse and with the broader finding that sexual trauma is more pathogenic than other interpersonal violence (Brewin, Andrews, & Valentine, 2000; Higgins & McCabe, 2000).

Low positive affect was associated with lower childhood social support, greater neglect, and greater caretaker dysfunction, consistent with our hypothesis and with previous findings indicating that childhood experiences are strong influences on later mental health (e.g., Green et al., 2010). Childhood social support was an especially robust predictor of positive affect, so interventions to foster a supportive environment for children may help to mitigate the deleterious effects of early adversity on positive affect. Along with family-focused interventions, at-risk children may benefit from mentoring, teacher-focused interventions, and nurturing extracurricular activities that grow social support outside of a family context.

Greater severity of abuse experiences, as measured by the number of incidents of abuse, predicted lower positive affect. Prior research has repeatedly found greater severity of traumatic experiences generally, and early adversity experiences specifically, to be associated with worse posttraumatic outcomes (Brewin et al., 2000; Cloitre et al., 2009; Higgins, 2004), and these results extend that line of research to a novel outcome—positive affect. However, the number of *non-abuse* traumas was not a significant predictor of positive affect in this study. It is unlikely that the other predictors in the model obscured the relationship between the number of traumas and positive affect, as the zero-order correlation between the two was comparatively small and not statistically significant. Instead, these results seem to suggest that childhood abuse experiences have a greater impact on positive affect than non-abuse childhood traumas or adult traumas. This finding is consistent with theory and research on the differentially greater impact of early interpersonal trauma (Cicchetti & Toth, 2005).

Potential mechanisms underlying the relationship observed in this study between low childhood social support, childhood sexual abuse, and low positive affect include disruption of attachment processes, emotion regulation difficulties and interactions with negative affect, and alteration of psychobiological state. Childhood maltreatment appears to be strongly associated with insecure attachments (Cicchetti & Toth, 2005). More insecurely attached individuals have been found to minimize positive experiences, and an avoidant attachment style has been associated with social anhedonia (Gentzler, Kerns, & Keener, 2010; Troisi, Alcini, Coviello, Nanni, & Siracusano, 2010), which would likely reduce how supportive individuals find social contacts and their likelihood of accessing social support. Furthermore, attachment difficulties have been associated with difficulty regulating negative affect (Cloitre, Stovall-McClough, Zorbas, & Charuvastra, 2008). These emotion regulation difficulties could interact with individuals’ ability to experience positive affect. Indeed, when individuals with PTSD were presented with typically positive stimuli (e.g., the image of a sunny day), they experienced significantly more negative affect than healthy controls (Frewen et al., 2012). This negative affect interfered with their ability to experience positive affect, contributing to individuals with PTSD experiencing less overall positive affect. In addition to these psychological consequences, attachment problems may have biological impacts. Psychobiological models have indicated that oxytocin, which is released during pleasurable social contact, resulting in feelings of reward, pleasure, and safety, plays a crucial role in positive affect and the endocrine response to stressors (Olff, 2012). Early negative emotional experiences and coping through over-modulation of affect are thought to produce lasting neurological alterations (Perry, Pollard, Blakely, Baker, & Vigilante, 1995), so childhood experiences of low social support may result in decreased oxytocin levels and concomitant reduced positive affect.

## Limitations

Limitations of the study include the potential for error in individuals’ recollection of childhood experiences and reports of low positive affect. In an experience sampling study of depressed patients, reports of anhedonia were found to be negatively biased (Ben-Zeev & Young, 2010). Additionally, current positive affect was not assessed so that it could be differentiated from lifelong experience of positive affect, and the reliability and validity of the positive affect items have not been established. This study was conducted with psychiatric inpatients, potentially limiting generalizability to less impaired populations; however, this study is notable for investigating positive affect in individuals with a range of psychiatric diagnoses and a range of childhood adversities. Future research would benefit from developing more extensive measures of lifetime positive affect and determining the relationship between measures of state positive affect (e.g., PANAS; Watson, Clark, & Tellegen, 1988) and lifetime positive affect as measured here.

## Conclusion

This study investigated the relationship between childhood adversity and positive affect in an adult psychiatric inpatient sample. Individuals who experienced both physical and sexual abuse as children reported lower lifetime experience of positive affect and lower lifetime capacity to experience positive affect than those with no abuse experience and those who experienced only one type of abuse. Sexual abuse severity was a particularly strong predictor of positive affect. Given the role of positive affect in psychological resilience, these results suggest that victims of multiple types of early adversity, especially those with sexual abuse experiences, may be at risk for psychological difficulties. Given the strong relationship between childhood social support and positive affect, interventions to foster social support may be useful in treating these at-risk populations.
